# Geospatial Analysis of Abiotic and Biotic Conditions Associated with Leptospirosis in the Klaten Regency, Central Java, Indonesia

**DOI:** 10.3390/tropicalmed9100225

**Published:** 2024-09-24

**Authors:** Dwi Sutiningsih, Dewi Puspito Sari, Cintya Dipta Permatasari, Nur Azizah Azzahra, Alfonso J. Rodriguez-Morales, Sri Yuliawati, Nine Elissa Maharani

**Affiliations:** 1 Department of Epidemiology and Tropical Diseases, Faculty of Public Health, Diponegoro University, Semarang 50275, Central Java, Indonesia; sriyuliawati@lecturer.undip.ac.id; 2 Master of Epidemiology, School of Postgraduate, Diponegoro University, Imam Bardjo S.H. Street, No.5, Semarang 50275, Central Java, Indonesia; nurazizahazzahra@untad.ac.id; 3 Public Health Study Program, Faculty of Public Health and Health Sciences, Veteran Bangun Nusantara University, Sukoharjo 57521, Central Java, Indonesia; sari.puspito.dp@gmail.com (D.P.S.); elissapanjimomo@gmail.com (N.E.M.); 4 Community Health Center of Traji Temanggung, Gembok, Traji, Parakan Sub-District, Temanggung Regency 56254, Central Java, Indonesia; cintya.dipta@gmail.com; 5 Masters of Climate Change and Clinical Epidemiology and Biostatistics Program, Universidad Cientifica del Sur, Lima 15307, Peru; arodriguezmo@cientifica.edu.pe; 6 Gilbert and Rose-Marie Chagoury School of Medicine, Lebanese American University, Beirut P.O. Box 36-5053, Lebanon

**Keywords:** leptospirosis, environmental factors, abiotic, biotic, spatial analysis, geographic information system (GIS)

## Abstract

The Klaten Regency, Central Java Province, Indonesia, is a leptospirosis endemic area. The purpose of this study is to spatially describe the abiotic and biotic environmental factors that contributed to the incidence of leptospirosis in the Klaten Regency in 2018. This was a descriptive observational with a cross-sectional approach conducted in the Klaten Regency, Central Java, in 2019 with 59 respondents. The results revealed that the percentage of abiotic environmental factors such as poor waste disposal facilities, poor gutter conditions, rivers < 200 m, and flooding history, namely 35.6%, 41.2%, 54.2%, and 6.8%, respectively. The highest leptospirosis cases occurred in April 2018, with 325 mm of rainfall, an average temperature of 27 °C, an average humidity of 82.3%, and an altitude of 100–200 MASL (79.7%). Meanwhile, biotic factors included rat nest existence (100%), having pets at risk (32.2%), and ≥three types of vegetation (79.7%). The main result confirmed that all leptospirosis cases had rat nests throughout the respondent’s house. This finding emphasizes the importance of rat pest control programs by establishing cross-sectoral collaboration with the Department of Agriculture and educating the public to also play a role in environmental cleanliness in controlling rats.

## 1. Introduction

*Leptospirosis* is a zoonotic disease that causes health problems throughout the world, especially in countries with tropical and subtropical climates and high rainfall [1,2]. Leptospirosis is caused by infection with pathogenic *Leptospira* species [3]. Leptospirosis disease could be transmitted from animals to humans or vice versa directly or indirectly [2,4]. Indirect transmission occurs when animals infected with Leptospira bacteria spread bacteria through the contaminated environment, such as soil, and water [4].

The annual estimation of leptospirosis globally was 1.03 million cases with 58,900 deaths. Southeast Asia is one of the endemic areas for leptospirosis [5,6]. One of the countries is Indonesia. Most areas in Indonesia have a tropical climate with high rainfall and humidity, so they have the potential to be a breeding ground for *Leptospira* bacteria [7].

Leptospirosis is endemic in several areas of Indonesia and has become a health problem for many years [8]. The mortality rate for leptospirosis in Indonesia is high, reaching 2.5–16.45% [9]. In 2018, Indonesia reported 895 cases of leptospirosis [10], with the highest contributed by Central Java (427, 47.7%) and 89 deaths (20.8%) [11]. The Klaten Regency is an endemic area of leptospirosis in Central Java Province. In 2018, Klaten Regency was ranked second in the number of leptospirosis cases in Central Java, with an Incidence Rate of leptospirosis in January–June of 4.18 per 100,000 population, exceeding the national target of 3 per 100,000 population [12].

Environmental factors are very influential in the incidence of leptospirosis. The interaction between the agent and the host occurs in the environment. Agent, host, and environment can produce habitats and bionomies that influence each other. Environmental conditions, including abiotic and biotic environmental factors, can also contribute to the incidence of leptospirosis [1,8,13,14].

Mapping the leptospirosis disease using a Geographic Information System (GIS) will provide an overview of the areas at risk. Therefore, this study aims to map the distribution of leptospirosis incidence and describe the abiotic and biotic environmental factors with the incidence of leptospirosis spatially in Klaten Regency, Central Java, Indonesia, in 2018.

## 2. Materials and Methods

### 2.1. Research Type and Design

This study uses descriptive observational research with a cross-sectional approach. It was conducted in Klaten Regency from June to August 2019.

### 2.2. Population and Research Sample

The population in this study were leptospirosis sufferers recorded in all public health centers and hospitals in the Klaten Regency in 2018, with as many as 67 patients. All members of the population were used as research samples with the inclusion criteria of residing in the Klaten Regency. While the exclusion criteria in this study were: did not want to be a respondent, the respondent moved house, and was unable to be found. Based on the inclusion and exclusion criteria, the number of research samples was 59 leptospirosis patients.

### 2.3. Data Sources, Data Processing, and Analysis

In this study, the authors use primary data and secondary data. The primary data in this study were collected through interviews and direct observation. Interviews and observations were conducted by filling out questionnaires and observation sheets related to biotic environmental factors. Interviews and observations were conducted after leptospirosis patients agreed to be respondents and signed the informed consent form. Researchers also took the coordinates of the locations of leptospirosis cases and altitude using an altitude measurement application. Secondary data in the form of rainfall data were obtained from the Klaten Public Works and Spatial Planning Office; temperature and humidity data were obtained from online data from the Meteorology and Geophysics Agency of Mlati Station; data on leptospirosis cases from January to December 2018 in Klaten Regency was obtained from Klaten District Health Service and digital SHP map data in Klaten Regency were obtained from the Agency for Regional Development and the Klaten Public Works and Spatial Planning Office. The data were then processed using software like Microsoft Excel Windows 7, IBM SPSS 25, and Arc GIS 10.3. Data processing includes editing, coding, entry, cleaning, and tabulating data.

**Figure 1:**
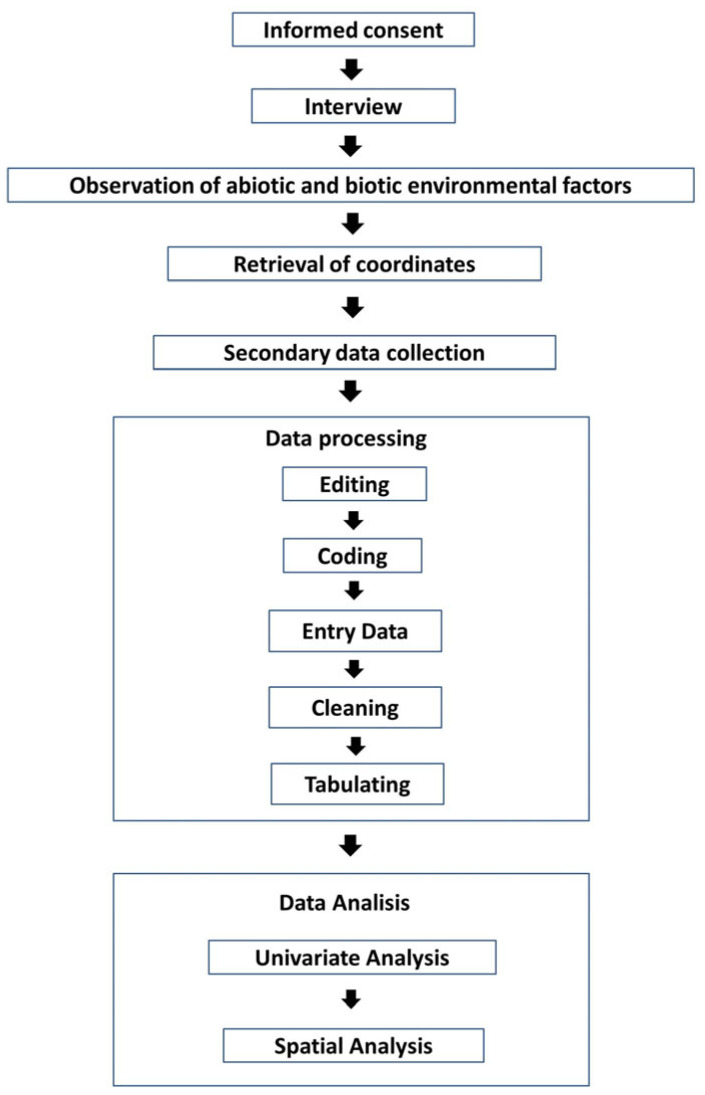
Flow Diagram of the Research.

Data analysis consisted of univariate analysis and spatial analysis. The univariate analysis included descriptions with frequency tables: abiotic environmental factors (presence of rat nests, waste disposal facilities, sewer conditions, working environmental conditions, presence of rivers, history of flooding, air temperature, humidity, rainfall, altitude), and biotic environmental factors (presence of risky pets, and type of vegetation). Spatial analysis was used to describe environmental factors (presence of rats, presence of risky pets, waste disposal facilities, gutter conditions, presence of rivers, history of flooding, vegetation, and altitude) with the incidence of leptospirosis in Klaten Regency with the Arc GIS 10.3 application. The results of the research were presented in the form of an overlay of abiotic environmental factors, including the presence of rat nests, waste disposal facilities, gutter conditions, history of flooding, altitude, and river buffers around the homes of leptospirosis sufferers. An overlay was also carried out on biotic environmental factors including the presence of at-risk pets and the type of vegetation. Apart from environmental factors, analysis of the distribution pattern of leptospirosis cases was carried out using autocorrelation analysis, which was calculated using the Moran’s Index method. The Moran Index in the case of a standardized spatial weighting matrix is −1 ≤ I ≤ 1. A value of −1 ≤ I < 0 indicates negative spatial autocorrelation, while a value of 0 < I ≤ 1 indicates positive spatial autocorrelation, and a Moran Index value of zero indicates no grouping. Positive spatial autocorrelation indicates the similarity of values from adjacent locations and tends to be clustered. Meanwhile, negative spatial autocorrelation indicates that nearby locations have different values and tend to spread out (Figure 1).

Spatial autocorrelation analysis studies disease distribution patterns and the incidence correlation in neighboring areas and provides recommendations for effective regional public health interventions to control infectious diseases, including leptospirosis [15]. Moran’s Index method, statistics for quantifying spatial autocorrelations of infectious diseases have been considerably employed [16,17].

### 2.4. Ethical Approval

This study has obtained ethical clearance from the Health Research Ethics Committee of the Faculty of Public Health, Diponegoro University, No. 246/EA/KEPK-FKM/2019. The respondents approved informed consent prior to the interviews and observations.

## 3. Results

### 3.1. Spatial Autocorrelation Results

**Figure 2:**
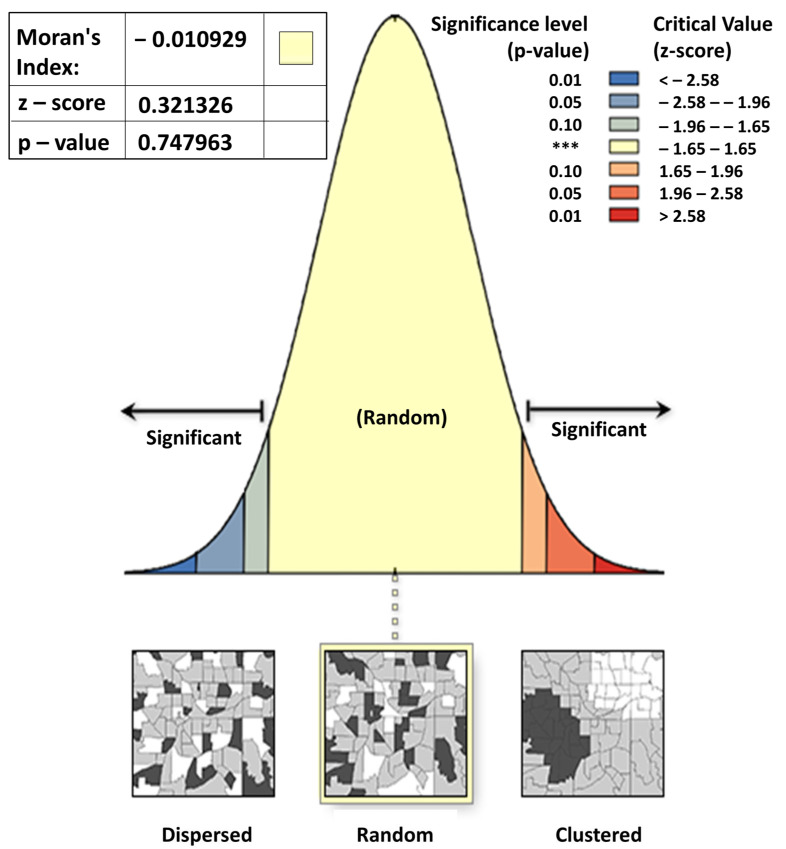
Spatial Autocorrelation Results.

Based on the statistical results of the Moran Index of −0.010929, the z-score value was 0.321326, and the *p*-value was 0.747963 (Figure 2), it can be concluded that the distribution pattern of leptospirosis in Klaten Regency in 2018 was random, which means that the position of cases in an area is not influenced by the position of other areas.

### 3.2. Abiotic Enviromental Factors

#### 3.2.1. Waste Disposal Facility

All respondents had a garbage dump (100%), around 93.2% of respondents had a trash can inside the house, but only a few had a trash can outside the house (40.7%). Besides, most of the respondents had garbage piled up (74.6%), waterproof trash cans (91.5%), and open trash cans (83.1%). Most of them threw the trash in the yard (59.3%) (Table 1).

The study results show that more respondents with good waste disposal facilities, 38 (64.4%) than those with poor disposal facilities, were 21 people (35.6%). The average distance between the trash can and the respondent’s house was 3.1 m (Table 1).

**Figure 3:**
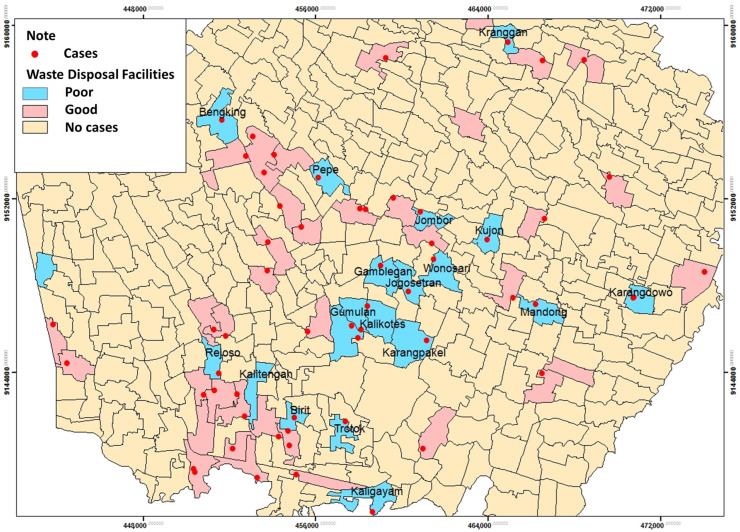
Distribution of Leptospirosis cases and village/urban village waste disposal facilities.

The spatial analysis of waste disposal facilities with leptospirosis incidence in the Klaten Regency in 2018 shows that respondents’ waste disposal facilities were poor in several respondent areas (35.6%). The spatial distribution of leptospirosis cases and waste disposal facilities showed that inadequate waste disposal facilities spread in 18 villages out of 52 villages and one urban village. These areas include: Kranggan, Bengking, Pepe, Jombor, Kujon, Wonosari, Gamblegan, Jogosetran, Gumulan, Kalikotes, Mandong, Karangdowo, Karangpakel, Rejoso, Kalitengah, Birit, Trotok, and Kaligayam (Figure 3).

#### 3.2.2. Sewer Condition

Table 2 shows that 34 people (57.6%) had sewers. There were 14 respondents with poor categories (41.2%) who had gutters. Most of the respondents had open sewers (91.2%), rats passing through the ditches (73.5%), and rat holes in the gutter (52.9%) (Table 2).

**Figure 4:**
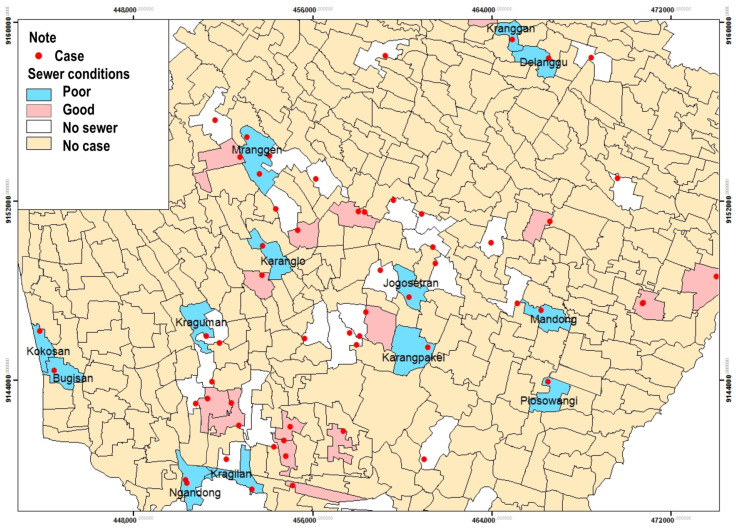
Distribution of Leptospirosis cases and sewer conditions by village/urban.

Based on the spatial analysis of sewer conditions and the incidence of leptospirosis in the Klaten Regency, it is shown that the presence of sewers can be a medium for the spread of leptospirosis disease. Based on the results of observations, it was shown that the rat holes in the ditches were in soil ditches that were not cemented. The condition of the sewers was poor in 13 villages out of 52 villages and one urban village, such as Kranggan, Delanggu, Mranggen, Karanglo, Jogosetran, Karangpakel, Mandong, Plosowangi, Kraguman, Kragilan, Ngandong, Bugisan, and Kokosan (Figure 4).

#### 3.2.3. The Existence of the River

Based on Table 3. reveals that as many as 32 respondents (52.2%) have a house close to the river <200 m from the respondent’s house (Table 3).

**Figure 5:**
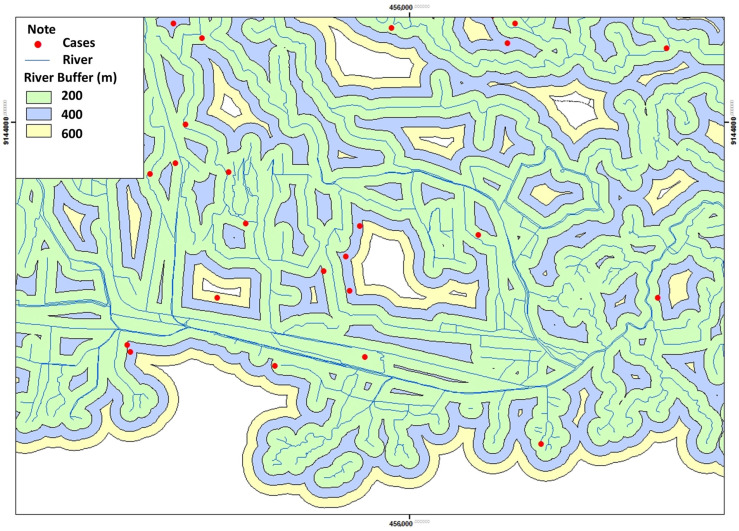
Distribution of River Presence with Leptospirosis Incidence.

The distribution of leptospirosis cases based on the presence of rivers in the Klaten Regency can be seen in Figure 5. Most of the respondents’ houses were located near the river at a distance of <200 m. The distance of the river < 200 m from the respondent’s house was spread over 31 villages and 1 urban village out of 52 villages and 1 urban village, which were Kokosan Village (52.8 m), Rejoso Village (3.7 m), Bakung Village (17.1 m), Ceporan Village (100 m), Towangsan Village (60.9 m), Kragilan Village (116.3 m), Ngandong Village (199.6 m), Plawikan Village (192.5 m), Nglinggi Village (72.7 m), Karanglo Village (106.8 m), Senden Village (39.4 m), Jebugan Village (75.5 m), Gumulan Village (25.5 m), Jomboran Village (172.9 m), Gamblegan Village (102.9 m), Kalikotes Village (136.4 m), Mayungan Village (27.1 m), Randulanang Village (140.3 m), Mlese Village (61.7 m), Canan Village (152 m), Trotok Village (79.3 m), Kaligayam Village (10 m), Karangpakel Village (17.8 m), Karangdowo Village (155.8–176.7 m), Kalitengah Village (39 m), Kadilanggon Village (164.2 m), Drono Village (104.2 m), Dalangan Village (20.9 m), Birit Village (96.8 m), Boto and Buntalan Villages (138.2 m) (Figure 5).

#### 3.2.4. Flood History

Based on Table 4 shows that fewer respondents experienced flooding, namely four respondents (6.8%), compared to respondents who did not experience flooding (91.5%) in 2018.

**Figure 6:**
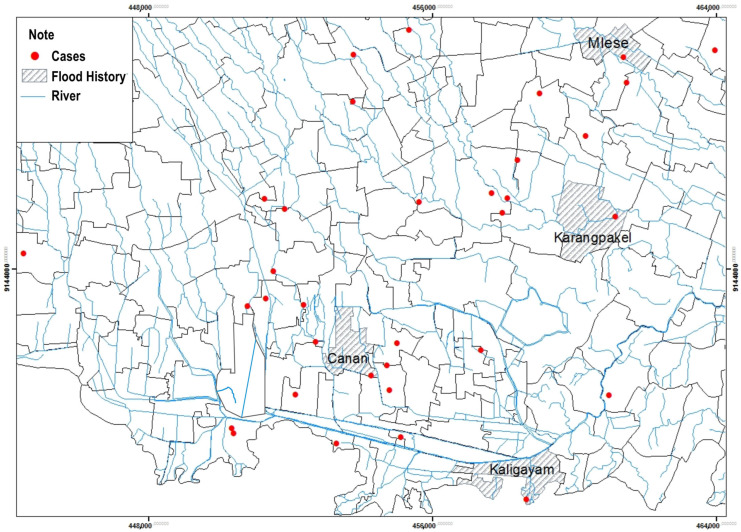
Distribution of Leptospirosis Cases and Flood History by Village/Urban Village.

A spatial analysis of flood history with the incidence of leptospirosis in the Klaten Regency shows that floods occurred in 2018 in several villages in the Klaten Regency. The history of flooding in the Klaten Regency within one year reached only a few numbers of respondents’ houses, spread over four villages from 52 villages and one urban village. Based on the map, the history of flooding was in the villages of Mlese, Canan, Kaligayam, and Karangpakel. Puddles of water during floods can be a medium for the spread of *Leptospira* bacteria (Figure 6).

#### 3.2.5. Rainfall, Air Temperature, Humidity, and Altitude

**Figure 7:**
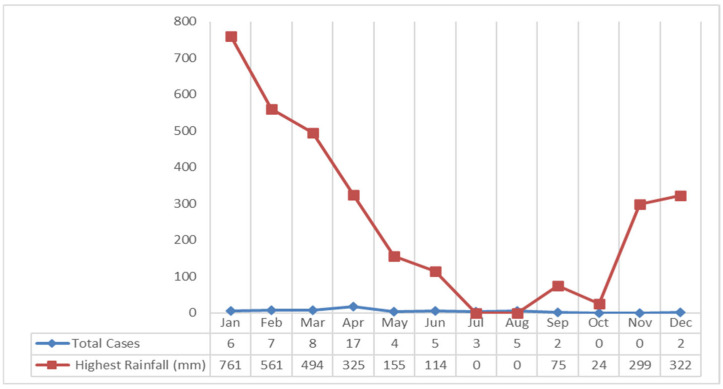
Graph of Highest Rainfall and Leptospirosis Cases in the Klaten Regency in 2018.

The study results showed that the rainfall in Klaten Regency tends to fall every month. The highest rainfall occurred in January 2018 at 761 mm, and the lowest occurred in October at 24 mm. If it is associated with leptospirosis cases, most cases occurred in April with 325 mm of rainfall which was included in the high category (Figure 7).

**Figure 8:**
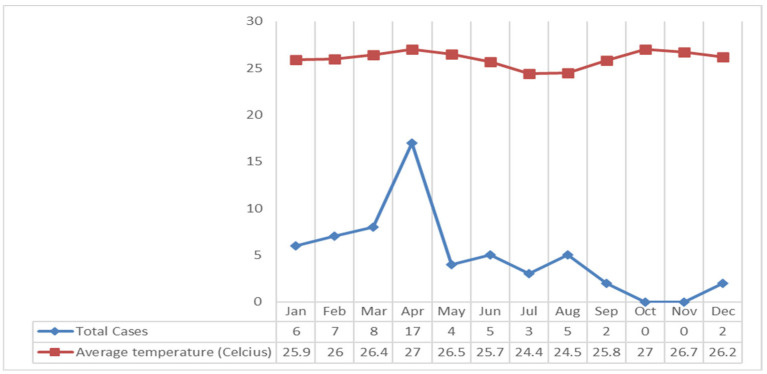
Graph of Air Temperature and Leptospirosis Cases in The Klaten Regency in 2018.

Based on Figure 8 describes that the air temperature in the Klaten Regency fluctuates. The highest air temperature occurred in April and October 2018 at 27 °C, and the lowest air temperature occurred in July at 24.4 °C. If it is associated with leptospirosis cases, most cases occurred in April, with an average air temperature of the highest in 2018 at 27 °C (Figure 8).

**Figure 9:**
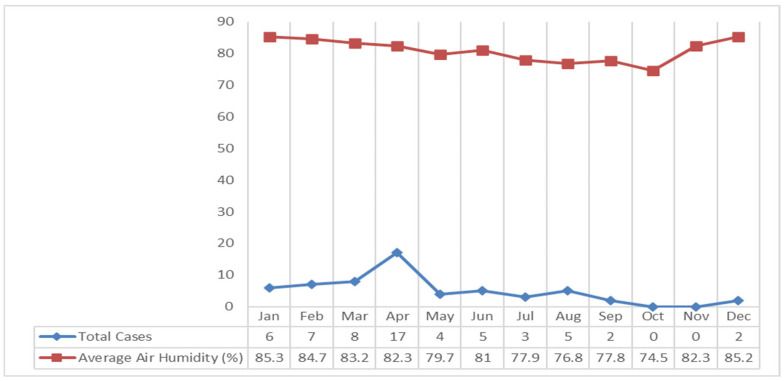
Graph of Air Humidity and Leptospirosis Cases in The Klaten Regency in 2018.

The average air humidity in Klaten Regency fluctuates. The highest average air humidity occurred in January 2018 at 85.3%, and the lowest in October at 74.5%. If it is associated with leptospirosis cases, most cases occurred in April with an average humidity of 82.3% (Figure 9).

Based on the results of the study, the most cases of leptospirosis were at an altitude of 100–200 M Above Sea Level (MASL), with as many as 47 respondents (79.7%) (Table 5).

**Figure 10:**
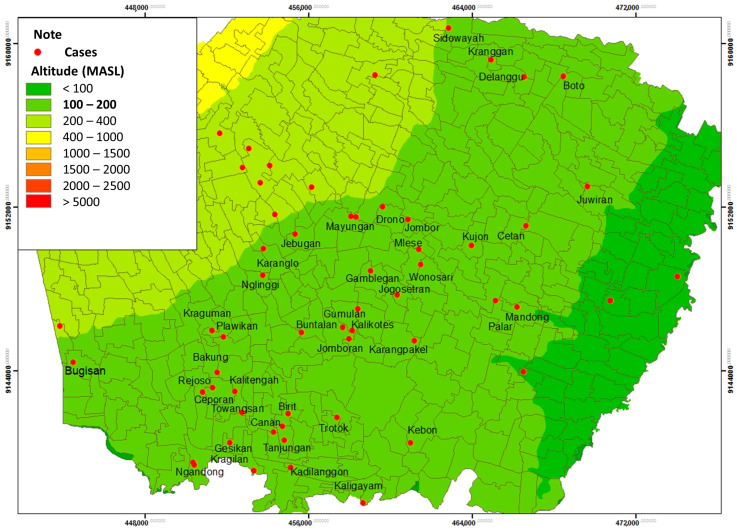
Distribution of Leptospirosis Cases Based on Altitude.

The spatial distribution shows that Leptospirosis cases in Klaten Regency spread at an altitude of <100 MASL, 100–200 MASL, and 200–400 MASL. Most respondents were located at an altitude of 100–200 MASL, spread over 40 villages from 52 villages and one urban village, which were Bugisan Village (186 MASL), Ngandong Village (139–145 MASL), Gamblegan Village (161 MASL), Wonosari Village (149 MASL), Kujon Village (148 MASL), Cetan Village (143 MASL), Jombor Village (188 MASL), Mlese village (150 MASL), Drono village (190 MAS), Mranggen village (270–307 MASL), Karanglo village (153 MASL), Jebugan village (143 MASL), Juwiran village (114 MASL), Boto village (151 MASL), Delanggu village (170 MASL), Kranggan (176 MASL), Sidowayah (205 MASL), Kragilan (131 MASL), Gesikan (144 MASL), Towangsan (149 MASL), Ceporan (158 MASL), Canan (144 MASL), Tanjungan (138 MASL), Kadilanggon (112 MASL), Kaligayam (148 MASL), Birit (143 MASL), Trotok (130 MASL), Kebon (129 MASL), Rejoso (160 MASL), Bakung (164 MASL), Plawikan (174 MASL), Kraguman (183 MASL), Buntalan (173 MASL), Jomboran (140–158 MASL), Karangpakel (134 MASL), Kalikotes (163 MASL), Gumulan (151 MASL), Palar (135 MASL), Mandong (177 MASL), and Nglinggi (200 MASPL) (Figure 10).

### 3.3. Biotic Environmental Factors

#### 3.3.1. Presence of Rats

Based on Table 6, there were nest rats all over the house respondents (100.0%). A total of 57 respondents (96.6%) had seen rats inside the home, and all respondents (100.0%) had ever seen a nest rat outside the home. Common types of rats seen by respondents were roof rats and wirok rats. Based on the observation in and around the respondents’ houses, some signs of rats included dirt, bites, and rat holes.

**Figure 11:**
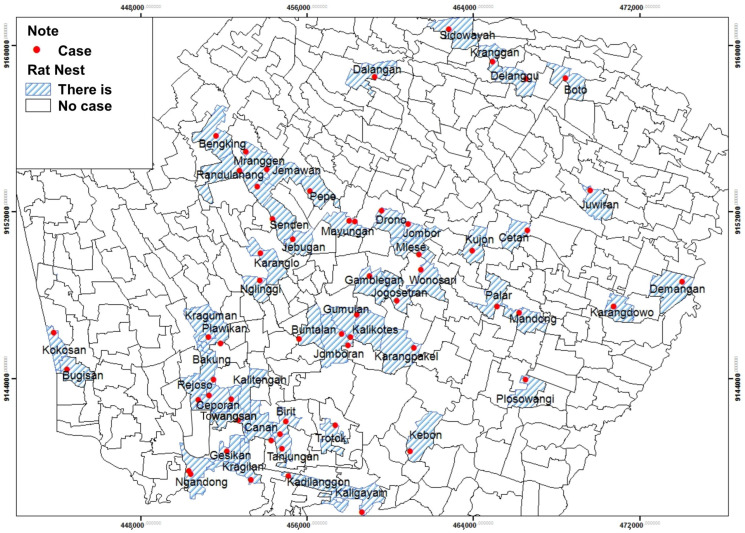
The Distribution of Leptospirosis Cases and the Presence of Rat’s Nests by Village/Urban Village.

Based on spatial analysis, nest rats exist throughout house respondents, which were in 52 villages and one urban village spread over 20 sub-districts. They were Bugisan, Kokosan, Rejoso, Bakung, Ceporan, Gesikan, Towangsan, Kragilan, Ngandong, Plawikan, Nglinggi, Karanglo, Senden, Jebugan, Kraguman, Gumulan, Jomboran, Gamblegan, Jogosetran, Kalikotes, Mayungan, Pepe, Jemawan, Mranggen, Randulanang, Bengking, Sidowayah, Kranggan, Delanggu, Juwiran, Mlese, Jombor, Cetan, Kujon, Plosowangi, Mandong, Palar, Wonosari, Canan, Birit, Trotok, Kaligayam, Kebon, Karangpakel, Karangdowo, Demangan, Kalitengah, Kadilanggon, Tanjungan, Drono, Dalangan, Boto villages, and Buntalan urban village (Figure 11).

#### 3.3.2. Presence of Pets at Risk

The Table 7 shows that most respondents did not have an animal pet at risk (67.8%) than those who had (32.2%). Cats were mostly found in the respondent’s houses (42.1%) (Table 7). 

**Figure 12:**
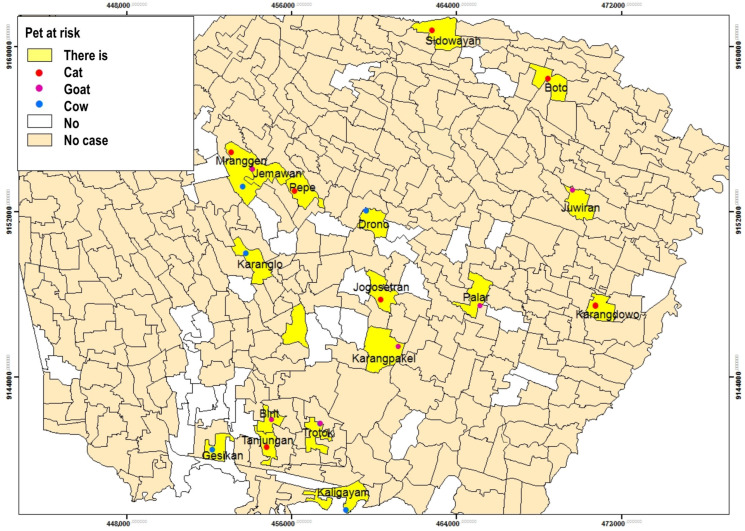
The Distribution of Leptospirosis Cases, and the Presence of Animals at Risk by Village/Urban Village.

Figure 12 reveals that pets are at risk in fewer respondents. The animal type pet risk owned by most respondents were cats, goats, and cows. The existence of animals at risk was spread over 16 villages and one urban village, such as Sidowayah Village, Boto Village, Mranggen Village, Jemawan Village, Pepe Village, Drono Village, Karanglo Village, Juwiran Village, Jogosetran Village, Palar Village, Karangpakel Village, Karangdowo Village, Trotok Village, Birit Village, Tanjungan village, Gesik Village, and Kaligayam Village (Figure 12).

#### 3.3.3. Vegetation Type

Table 8 reveals that the most vegetation type around house respondents was tree shade (93.2%). Respondents mostly had ≥ three vegetations (79.7%). The average distance between vegetation and house respondents was 4.5 m (Table 8). 

**Figure 13:**
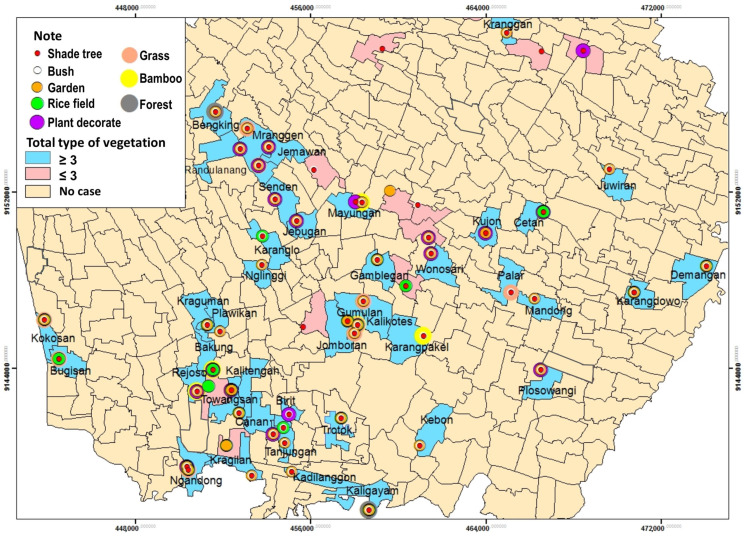
Distribution of Leptospirosis Cases and Types of Vegetation by Village.

Based on Figure 13, ≥3 vegetations types spread over 42 villages from 52 villages and 1 urban village such as Bugisan Village with 3 types vegetation, Kokosan with 5 types vegetation, Rejoso with 4 types vegetation, Bakung with 4 types vegetation, Towangsan with 4 types vegetation, Kragilan with 3 types vegetation, Ngandong with 5 types vegetation, Plawikan with 3 types vegetation, Nlinggi with 3 types vegetation, Karanglo with 3 types vegetation, Senden with 4 types vegetation, Jebugan with 4 types vegetation, Kraguman with 4 types vegetation, Gumulan with 6 types vegetation, Jomboran with 4 types vegetation, Gamblegan with 4 types vegetation, Kalikotes with 4 types vegetation, Mayungan with 5 types vegetation, Jemawan with 4 types vegetation, Mranggen with 4 types vegetation, Randunang with 4 types vegetation, Bengking with 4 types vegetation, Kranggan with 3 types vegetation, Juwiran with 3 types vegetation, Mlese with 4 types vegetation, Cetan with 3 types vegetation, Kujon 3 types vegetation, Plosowangi with 4 types vegetation, Mandong with 3 types vegetation, Palar with 3 types vegetation, Wonosari with 4 types vegetation, Canan with 4 types vegetation, Birit with type vegetation, Trotok with 5 types vegetation, Kaligayam with 6 types vegetation, Kebon with 3 types vegetation, Karangpakel with 7 types vegetation, Karangdowo with 3 types vegetation, Demangan with 4 types vegetation, Kalitengah with 4 types vegetation, Kadilanggon with 3 types vegetation, and Tanjungan with 3 types vegetation (Figure 13).

## 4. Discussion

Recent research shows that most of the respondents have good disposal facilities. This result is similar to a previous study that showed 81.1% of the residential area in Si Sa Ket, Thailand, had garbage disposals [18]. However, the majority of the cases had garbage piled up inside their house. This condition led to rats in the trash in some of their homes. Poor house sanitation is a risk factor for leptospirosis, and outbreak [4,19]. Besides, the results of this study indicate that the average distance between the outside garbage and the respondent’s house is 3.1 m. The increasing rat population can be affected by garbage around the house [4].

In various studies, the spread of leptospirosis has been linked to poor garbage disposal and sanitation. In Saint Lucia, poor garbage disposal was identified as a significant factor, contributing to 66.0% of the cases [20]. Similarly, in an urban South Indian city, inefficient garbage disposal was a major epidemiological risk factor, contributing to 95.5% of the cases detected [21]. In Semarang City, spatial analysis revealed that almost all regions with leptospirosis cases had garbage disposal facilities at risk (95.5%) [22]. Poor rubbish dumps are a risk factor for leptospirosis because the intermediary vector of Leptospira bacteria, particularly rats, thrives in places with piles of rubbish [23].

Based on interviews and observations, many respondents have open gutters. Rats often pass through sewers, with rat holes in the gutters. The above conditions are closely related to risk factors for leptospirosis. Mice that pass through water can spread *Leptospira* bacteria through rat urine. Sewers allow rats to migrate to other areas for shelter and food, including roads to enter people’s homes through drainage [24]. The research in the Kodagu district of southern India revealed that proximity to an open sewer was associated with leptospirosis (*p*-value = 0.02) with adjusted Odds Ratio [aOR] = 4.9 (CI: 1.2–19.1) [25].

The proximity of residences to open sewers has been identified as a significant risk factor for both primary and secondary infections of leptospirosis. According to [26], living near an open sewer was significantly associated with an increased risk of primary infection, with an odds ratio (OR) of 0.95 (CI: 0.91–0.99) for each 1-m increase in distance from the sewer. A study in six urban slums in Brazil found that soils surrounding conventionally closed sewers were three times less likely to contain pathogenic Leptospira and had a six times lower load of the pathogen compared to those near open sewers [27]. Living in flood-risk regions with open sewers was associated with a higher risk of acquiring Leptospira antibodies, with a prevalence ratio (PR) of 1.42 (95% CI 1.14–1.75) [28]. Proximity to an open sewer was an independent risk factor for acquiring leptospirosis, with a matched OR of 5.15 (95% confidence interval [CI] = 1.80–14.74) [29].

Based on the study’s results, it was shown that respondents with poor sewer conditions were almost half of the total respondents. Sewer conditions are a risk factor for leptospirosis infection [30]. In contrast, a study in the highlands of Ponorogo Regency, Province of East Java, Indonesia, showed that 21 (75%) cases had eligible sewerage [4].

Based on the results of the study showed that most cases of leptospirosis were around rivers with river buffers < 200 m. The closest distance from the river is 3.7 m. Based on observations, many rat holes were seen on the river bank. When the river water rises, the rats come out of the hole and enter people’s houses. Based on the study results, although the respondents’ location was close to the river, only a small number of respondents had experienced flooding and were in the respondent’s area. The overflow of river water found in a small number of respondents only flooded the streets around the house. However, the puddle can potentially spread *Leptospira* bacteria in the environment. Many leptospirosis outbreaks occur during heavy rains and floods [31]. The existence of conditions after a flood allows people to come into direct contact with polluted water and is favorable for developing infection [30]. Research in Kalitengah village, Wedi District, Klaten Regency, found that two rats of the *Rattus tanezumi* species were positive for Leptospira bacteria out of 17 rats caught. *Rattus tanezumi* habitats close to humans in residential areas can be a source of Leptospira transmission and can spread to humans and other environments [32].

The highest number of leptospirosis cases occurred in April, with 325 mm of rainfall in the high category. The high rainfall in Klaten Regency led to puddles around the house, which caused the gutters to overflow and inundate the surrounding streets. Based on data on average temperature and humidity each month, the average temperature is 27 °C in April. The average air temperature is close to the optimal temperature for growth of leptospirosis, which is 28–30 °C [33]. A recent study revealed that the average humidity is 82.3%. The average air humidity supports the growth of *Leptospira* bacteria outside its host. Similarly, a study in another endemic area in Kandy district, Sri Lanka in 2008–2015 showed that peaks in wet days per week, days with rainfall over 100 mm per week, minimum temperature, average temperature (25 °C), and average humidity (83.7%) were followed by peaks in leptospirosis incidence after lags of 2, 3, 13, 20, and 1 weeks, respectively [34,35]. The increases in the cases have been correlated with high rainfall, temperature, and relative humidity that support the survival of rodents and rodent densities, and the bacteria [31,34,36,37,38].

Based on spatial analysis, nests were found in all respondents’ houses. Respondents often see roof rats and shrew types in the house, while sewer and wirok rats are outside. This study was supported by revealing that most respondents had seen rats at home, with 62.7% [39]. The rat’s existence in the house was associated with leptospirosis incidence with a *p*-value of 0.050 [4]. Exposure to carrier rodents was related to the risk of disease transmission [40].

A study conducted in Pati in 2019 stated that the presence of rats was proven to be a risk factor for leptospirosis. The presence of rats in and around the house has a 4.51 times greater risk of infecting leptospirosis than the absence of rats [41]. The study results show that fewer respondents have pets than those who do not. Based on the spatial analysis, at-risk pets are present only in several areas. The most common pet owned by the respondents is a cat. A study conducted in Southern Chile in 2014 conveyed that Leptospira seropositive was found in cats. Animal habitat characteristics and some agricultural activities carried out by cat owners were risk factors associated with seropositivity [42].

Cats in all respondents’ homes were allowed to roam inside and outside. Other pets owned by respondents are goats and cows. Cows and goats are in the stable. Sometimes it is placed in the garden and tied so that it does not wander into the streets or residents’ houses. This study is in line with previous study showing that almost a third of cases had pets or livestock [39]. The study previously revealed that livestock ownership, cattle ownership, and the distance from the house to the cowshed were associated with the incidence of leptospirosis with *p*-values of 0.004, 0.010, and 0.024, respectively. The odds Ration calculation of livestock ownership was 13.830 (with a 95% CI of 1.702–112.382) [4]. Infected animals can spread leptospirosis through direct urine, other non-salivary body fluids, or contaminated soil or water [4,43]. Having livestock or pets is one of the factors related to disease transmission [40,44]. Sharing habitat between animals allows leptospirosis transmission [44].

The results showed that the majority of respondents who live around their houses have three types of vegetation, with shade trees being the most common vegetation. Vegetation is also a habitat for rodents, for example, bushes and rice fields. Vegetation availability in the yard also allows rodents to enter the house through branches or twigs adjacent to the house by climbing [22]. Abundant vegetation positively influences rodent abundance by providing food, a suitable breeding environment, and shelter [45]. A lot of vegetation cover can increase the persistence of the Leptospira spp.-free life stage because it is associated with lowering the ambient temperature, solar radiation, and increasing humidity [38,45].

Most respondents’ dominant environmental factors in Klaten Regency as leptospirosis endemic areas were the presence of rat nests, ≥three vegetation types, and river availability < 200 m. Other studies in other endemic areas in Sleman and Ponorogo supported this study [46,47]. Research results in Sleman District Regional Province Special Yogyakarta showed that most leptospirosis occurred in locations with the presence of rats (85.2%), vegetation (100%), and trenches/ditches around the house (47.5%) [47]. Study in Ponorogo Regency, Province of East Java, Indonesia revealed the majority of leptospirosis cases had the presence of rats in the house (92.9%), The presence of rats around the house (78.6%), and vegetation (82.1%) [4].

This research had been attempted and carried out following scientific procedures, but there were still limitations. Firstly, air temperature and humidity data were obtained from the Yogyakarta Mlati station. Klaten Regency did not have a temperature and humidity monitoring station. The nearest air temperature and humidity monitoring stations are in Karanganyar and Adi Sumarmo Airport. Because of the incompleteness of the data in the nearest stations, the authors used Mlati station data under the direction of the Semarang Meteorology and Geophysics Agency. Besides, the abiotic data collection used secondary data, and the research design used descriptive study. Furthermore, control of potential confounders such as socio-demographic factors, socioeconomic factors, healthcare access, and population density was not included in this research. In addition, not all cases of leptospirosis obtained from the Klaten District Health Service were successfully included in the study; of the 67 cases, 59 were successfully visited, interviewed, and observed. Further research requires more samples and control samples to generalize the more accurate findings.

This research was conducted in 2019, and there is the possibility that the environmental conditions of respondents have changed, whether they improve or get worse. However, Klaten Regency still had many cases with the highest prevalence and IR of leptospirosis in Central Java, namely 80 cases and 6/100,000 population, respectively, in 2022 [11].

## 5. Conclusions

Leptospirosis continues to be a significant public health problem in multiple tropical countries, leading to significant morbidity and mortality and then requiring better study and control approaches [32,33,34].

In conclusion, the percentage abiotic factors included poor waste disposal facilities, poor gutter conditions, presence of rivers <200 m, and history of flooding are 35.6%, 41.2%, 54.2%, and 6.8%, respectively. Most Leptospirosis occurred in April, with the highest rainfall of 325 mm, air temperature of 27 °C, humidity 82.3%, and altitude between 100–200 MASL. Based on biotic environmental factors, the presence of rat nests was found in all respondents’ houses (100.0%), respondents who had pets at risk (32.2%), and the type of vegetation around the respondent’s house three types (79.7%). Implementing the One Health approach is necessary to prevent and control zoonosis, including Leptospirosis incidence, through cross-sectoral collaboration, for example, in controlling rats in the Klaten Regency. The recommendations for further studies are using case-control as an analytical study design and direct measurements such as rainfall and altitude data, temperature, and humidity to produce more accurate data.

## Figures and Tables

**Table 1:** Waste disposal facilities.

Waste Disposal Facilities	Frequency	Percentage (%)
Garbage dump		
Yes	59	100.0
Not	0	0
Where is the trash thrown?		
Yard	35	59.3
River	8	13.6
Ricefield	1	1.7
Temporary waste storage	15	25.4
In house		
Trash can		
Yes	55	93.2
Not	4	6.8
Garbage piled up		
Yes	44	74.6
Not	11	18.6
Do not have	4	6.8
Rats in the trash		
Yes	19	32.2
Not	36	61.0
Do not have	4	6.8
Waterproof trash can		
Yes	54	91.5
Not	1	1.7
Do not have	4	6.8
Open trash can		
Yes	49	83.1
Not	6	10.2
Do not have	4	6.8
Trash can type		
Plastic trash can	29	49.2
Bucket	6	10.2
Crackle plastic	17	28.8
Bag	2	3.4
Cardboard box	1	1.7
Do not have	4	6.8
Outside house		
Trash can		
Yes	24	40.7
Not	35	59.3
Garbage piled up		
Yes	16	27.1
Not	10	16.9
Do not have	33	55.9
Rats in the trash		
Yes	19	32.2
Not	7	11.9
Do not have	33	55.9
Waterproof trash can		
Yes	14	23.7
Not	10	16.9
Do not have	35	59.3
Open trash can		
Yes	24	40.7
Do not have	35	59.3
Trash can type		
Plastic trash can	1	1.7
Bucket	4	6.8
Crackle plastic	1	1.7
Bag	8	13.6
Cardboard box	9	15.3
wooden basket	1	1.7
Do not have	35	59.3
Condition		
Poor	21	35.6
Good	38	64.4

**Table 2:** Sewer condition.

Sewer	Frequency	Percentage (%)
Sewers		
Yes	34	57.6
No	25	42.4
Open sewer condition		
Yes	31	91.2
Not	3	8.8
Rat hole in sewer		
Yes	18	52.9
Not	16	47.1
Rats pass sewer		
Yes	25	73.5
Not	9	26.5
Trash in sewer		
Yes	6	17.6
Not	28	82.4
Sewer water overflowed into the street		
Ever	15	44.1
Never	19	55.9
Sewer water is pooling		
Yes	12	35.3
Not	22	64.7
Condition		
Poor	14	41.2
Good	21	61.8

**Table 3:** River Existence.

The Existence of the River	Frequency	Percentage (%)
<200 m	32	54.2
200–400 m	17	28.8
>400 m	10	16.9
Total	59	100.0

**Table 4:** Flood History.

Flood History	Frequency	Percentage (%)
Yes	4	6.8
Not	55	93.2
Total	59	100.0

**Table 5:** Altitude of Place.

Altitude of Place (MASL)	Frequency	Percentage (%)
<100	3	5.1
100–200	47	79.7
200–400	9	15.3
400–1000	0	0.0
1000–1500	0	0.0
1500–2000	0	0.0
2000–2500	0	0.0
>2500	0	0.0
Total	59	100.0

**Table 6:** Mice’s Nest in Respondent’s House.

Existence of the Rat’s Nest	Frequency	Percentage (%)
Nest rat Existence		
yes	59	100.0
no	0	0.0
Nest rat inside house		
yes	57	96.6
no	2	3.4
Ever see nest rat around house		
yes	59	100.0
no	0	0.0

**Table 7:** Pet Place at risk.

Pets-At Risk	Frequency	Percentage (%)
Presence of pets-at risk		
yes	19	32.2
no	40	67.8
Pet Type		
Cat		
yes	8	42.1
no	11	57.9
Goat		
yes	6	31.6
no	13	68.4
Cow		
yes	6	31.6
no	13	68.4

**Table 8:** Types of Vegetation.

Vegetation Type	Frequency	Percentage (%)
Bush		
There is	43	73.9
No	16	27.1
Tree Shade		
There is	55	93.2
No	4	6.8
Ricefield		
There is	21	35.6
No	38	64.4
Grass		
There is	9	15.3
No	50	18.7
Plant Decorate		
There is	1.9	32.2
No	4.0	67.8
Garden		
There is	4.1	69.5
No	1.8	30.5
Tree Bamboo		
There is	5	8.5
No	54	91.5
Forest		
There is	2	3.4
No	57	96.6
Total		
≥3	47	79.7
<3	12	20.3

## Data Availability

Data will be made available on request.
